# Somatoform, Functional, and Secondary Psychiatric Disorders: Why the Distinction Matters

**DOI:** 10.1192/bjo.2026.11898

**Published:** 2026-06-30

**Authors:** Caroline Tretton, William Hennessy

**Affiliations:** Bodmin Hospital - Cornwall Partnerships NHS Foundation Trust, Bodmin, United Kingdom

## Abstract

**Aims:**

Distinguishing primary functional disorders from psychiatric illness arising secondary to chronic physical symptoms remains a significant clinical challenge, particularly when structural pathology is absent. Failure to make this distinction risks premature psychological attribution, diagnostic overshadowing, and invalidation of patient experience. Although ICD-11 offers updated frameworks for understanding disorders at the brain–body interface, their application to complex presentations requires careful clinical judgement to avoid misinterpretation of psychiatric risk.

**Methods:**

Case Report

**Results:**

We present a case that illustrates this diagnostic tension. A 48-year-old woman with high premorbid functioning and no prior psychiatric history developed a progressive multisystem physical illness following international travel. Symptoms included gastrointestinal dysmotility, bladder dysfunction, autonomic instability, and sleep disruption. Despite extensive investigation, no unifying structural or neurodegenerative diagnosis was identified.

As the physical illness persisted, the patient developed a severe depressive syndrome with suicidality. Notably, affective symptoms emerged only after the onset of physical decline and fluctuated in parallel with physiological distress, rather than following an autonomous psychiatric course.

This case highlights the importance of distinguishing primary functional disorders from psychiatric illness that is reactive to chronic physical dysfunction. Contemporary models conceptualise functional conditions as disorders of brain–body interaction, characterised by altered interoceptive processing and maladaptive predictive mechanisms. In such cases, these processes are central to symptom generation and represent appropriate targets for psychiatric intervention.

By contrast, secondary psychiatric illness arises in response to prolonged physical uncertainty, loss of bodily predictability, and erosion of trust in physiological functioning. In this context, the depressive syndrome does not account for the physical symptoms but develops as a consequence of them. Temporal sequencing is therefore a key diagnostic marker. Failure to recognise this distinction risks premature psychological attribution, erosion of the therapeutic alliance, and misinterpretation of suicide risk. In such cases, suicidality is often driven by existential distress related to persistent physical suffering and fear of irreversible decline, rather than affective pathology alone.

**Conclusion:**

Not all distress associated with unexplained physical symptoms is somatoform. Psychiatry’s role is not to resolve medical uncertainty through psychological explanation, but to locate distress accurately within the patient’s causal narrative. Careful formulation, grounded in phenomenology and temporal sequencing, is essential for ethical practice and effective risk management - and in cases such as these can prove to be life saving

